# Haloperidol-induced painless legs and moving toe syndrome in a schizophrenia patient: a case report

**DOI:** 10.2144/fsoa-2023-0207

**Published:** 2024-05-15

**Authors:** Mugundan U Maheshwari, Natarajan Shanmugasundaram, Karthik Sankar, Mahendran Sekar, Ling S Wong, Sarvesh Sabarathinam, M Yasmin Begum, Srikanth Jeyabalan

**Affiliations:** 1 Department of Pharmacy Practice, Sri Ramachandra Faculty of Pharmacy, Sri Ramachandra Institute of Higher Education & Research (DU), Porur, Chennai, 600 116, Tamil Nadu, India; 2 Department of Psychiatry, Sri Ramachandra Medical College & Research Institute, Sri Ramachandra Institute of Higher Education & Research (DU), Porur, Chennai, 600 116, Tamil Nadu, India; 3 School of Pharmacy, Monash University Malaysia, Bandar Sunway, Subang Jaya 47500, Selangor, Malaysia; 4 Faculty of Science, Technology, Engineering and Mathematics, INTI International University, Nilai, Negeri Sembilan 71800, Malaysia; 5 Drug Testing Laboratory (DTL), Interdisciplinary Institute of Indian System of Medicine (IIISM), SRM Institute of Science and Technology, Kattankulathur, Chennai, Tamil Nadu-603203, India; 6 Department of Pharmacuetics, College of Pharmacy, King Khalid University, Abha, Saudi Arabia; 7 Department of Pharmacology, Sri Ramachandra Faculty of Pharmacy, Sri Ramachandra Institute of Higher Education and Research (DU), Porur, Chennai-600116, Tamilnadu, India

**Keywords:** haloperidol, painless legs, tardive dyskinesia

## Abstract

Painless legs and moving toe syndrome (PoLMT) is a rare syndrome characterized by involuntary movements of the toe without pain. The exact etiology of the patient's PoLMT is unknown. We present a case of PoLMT in 45-year-old woman with a history of haloperidol intake for 10 months. Haloperidol was discontinued, and aripiprazole (15 mg) was initiated. After this switch, a reduction in movement was observed in the third and fourth toes; however, the second toe showed no discernible change.

## Plain language summary

Painless Legs and Moving Toe Syndrome (PoLMT) is a rare condition in which the toe moves on its own without any pain. No one knows for sure what causes PoLMT in patients. In this case report, we discuss a 45-year-old woman with PoLMT who was taking a drug called haloperidol for 10 months prior to their visit to hospital. Another drug, aripiprazole, was started after haloperidol was stopped. It was noticed that the third and fourth toes moved less after this switch in medication, but no change was noticed in the second toe.

## Abstract (plain language summary)

PoLMT is a rare condition characterized by continuous and involuntary toe movements that occur without pain.

It is hypothesized that PoLMT might be a rare side effect of the patient's antipsychotic treatment since she had been on haloperidol 5–10 mg daily for 10 months prior to presentation. Tab. haloperidol 10 mg, which can decrease dopamine levels and may lead to movement disorders.

Following a two-week discontinuation of Tab. haloperidol, we observed a modest improvement in PoLMT.

Painless legs and moving toe syndrome (PoLMT) is a rare condition characterized by continuous and involuntary toe movements that occur without pain. This syndrome can affect individuals of any age group [1,2]. This condition is a variant of the syndrome characterized by painful legs and moving toes [3]. The etiology and mechanism of PoLMT remain unclear [4]. Some mechanisms are speculated due to alterations in afferent sensory information and peripheral nerve damage due to the development of dystonia [5,6]. Additionally, recent research has observed that PoLMT may be a rare manifestation of parkinsonism [7,8]. The disease has been observed in individuals spanning a broad age spectrum, including an 11-year-old girl and an 86-year-old patient. To date, only a limited number of reports have been documented. The initial publication on the subject occurred in 1993, wherein the condition was referred to as PoLMT.

## Clinical case

A 45-year-old woman was admitted to a tertiary care hospital with a history of talking to herself, hearing voices, being suspicious of her husband, sleep disturbances, anger and irritability. The patient has been diagnosed with schizophrenia for the past 18 months and is currently prescribed a daily dose of 10 mg of haloperidol, which was initially started at 5 mg. The individual had a BMI of 30.3 kg/m^2^, indicating obesity. They exhibited average psychomotor function and reported no substance use. During her personal history disclosure, the patient reported experiencing abnormal, painless, continuous, involuntary movement in the second, third and fourth toes of her left foot for the past 6 months. She was unable to stop the movement and exhibited no symptoms of bradykinesia, rigidity or tremors. There was no evidence of prior brain injury or trauma, and there was no familial history of movement disorders. This patient had no movements in her toes during sleep and no urges to move her legs or toes. The movements have minimal influence on her daily activities due to the emotional distress and concern they cause her. There were no indications of any further neurological abnormalities, such as sensory difficulties, reduced tendon reflexes, weakness or muscular atrophy. Both computerized tomography (CT) and magnetic resonance imaging (MRI) scans showed no evidence of lesions, tumors, infarctions or hemorrhages. The MRI scan of the entire spinal cord did not indicate any signs of compression. Nerve conduction studies and blood examinations, including Hepatitis Virus (HBV and HCV), renal, liver, and thyroid function tests, were found to be normal. Hemoglobin A1c (HbA1c) level was 6.1%, vitamins B1 and B12, ceruloplasmin and serum iron levels were within the normal range. Additionally, a lumbar puncture was conducted and no abnormalities were observed in the report.

The precise cause of the patient's PoLMT remains uncertain, despite considering factors such as the absence of pain and other clinical indicators. It is hypothesized that PoLMT might be a rare side effect of the patient's antipsychotic treatment since she had been on haloperidol 5–10 mg daily for 10 months prior to presentation. According to the literature, the patient began treatment with oral clonazepam at a dosage of 0.5 mg/day, which was subsequently increased to 1 mg/day. Gabapentin at a dosage of 300 mg administered three times daily was prescribed for the patient with PoLMT. However, there was no improvement in the movements. Haloperidol was discontinued for a period of 2 weeks, and a decrease in movement was noted in the third and fourth toes, while no noticeable change was observed in the second toe (video 1: before dechallenge with haloperidol, and video 2: after dechallenge with haloperidol). The improvement in the movement of the third and fourth toes was noticed only after being dechallenged with haloperidol, which was clearly observed in video 2. Before the dechallenge, all three toes had movement, which led us to speculate that haloperidol might induce PoLMT. Then they switched to an atypical antipsychotic (aripiprazole 15 mg). The mechanism behind this switch therapy is that haloperidol has a strong antagonistic effect on the D2 receptors; in contrast, aripiprazole is a newer atypical antipsychotic that displays a unique pharmacological profile, including partial D2 agonism, but both antipsychotics act via the D2 receptor. On the other side, clozapine induces fewer EPS than other antipsychotic medications, especially less akathisia and tremor, and usually no dystonia or rigidity. In patients with dyskinetic movements induced by other antipsychotics, clozapine may reduce or even remove dyskinesia. The low level of EPS with clozapine may be linked to its receptor-binding profile because during clozapine treatment, the level of D_2_ receptor blockade is too low to induce EPS, but due to obesity risk, this patient was prescribed Aripiprazole. The patient was discharged upon her request and was prescribed the following medications upon discharge: a daily dose of 15 mg of aripiprazole, a daily dose of 1 mg of clonazepam, and a daily dose of 20 mg of pantoprazole. Written informed consent was obtained from the patients for inclusion in this case report.

## Discussion

To the best of our knowledge, this is the first instance of PoLMT syndrome linked to haloperidol in schizophrenia patients. The etiology of both PLMT and PoLMT is still unclear, and it is assumed that PoLMT is a variant of PLMT [3]. From the previous case reports and studies, peripheral nerve involvement, central nervous system (CNS) involvement, Wilson's disease, cerebral infraction, brain tumor, and spinal cord compression have been reported to be associated with PoLMT syndrome. For treatment, quetiapine, gabapentin, or low doses of clonazepam were reported to be effective [4]. It is usually associated with peripheral nerve lesions such as radiculopathy and peripheral neuropathy [7]. A case report from Kawajiri S *et al.* suggests that PoLMT syndrome may be a rare manifestation of the antipsychotic medication haloperidol, and in contrast, the administration of rotigotine, a dopamine agonist, led to a significant improvement in PoLMT. Conversely, ropinirole, another dopamine agonist, had a detrimental effect on PoLMT. Rotigotine exhibits higher affinity for the dopamine D1 receptor compared with ropinirole [^8-10^].

## Conclusion

In this instance, we found no obvious neurological abnormalities, and the patient is on regular Tab. haloperidol 10 mg, which can decrease dopamine levels and may lead to movement disorders. Following a 2-week discontinuation of Tab. haloperidol, we observed a modest improvement in PoLMT. This finding lends support to our hypothesis that PoLMT may be a rare adverse effect of haloperidol. Further research is required to investigate the potential occurrences of these rare situations in order to substantiate this hypothesis.
